# Genetic Parameters and the Impact of Off-Types for *Theobroma cacao* L. in a Breeding Program in Brazil

**DOI:** 10.3389/fpls.2017.02059

**Published:** 2017-12-01

**Authors:** Ashley DuVal, Salvador A. Gezan, Guiliana Mustiga, Conrad Stack, Jean-Philippe Marelli, José Chaparro, Donald Livingstone, Stefan Royaert, Juan C. Motamayor

**Affiliations:** ^1^Mars Inc., Miami, FL, United States; ^2^Horticultural Sciences Department, University of Florida, Gainesville, FL, United States; ^3^School of Forest Resources and Conservation, University of Florida, Gainesville, FL, United States; ^4^Mars Center for Cocoa Science, Itajuípe, Brazil

**Keywords:** cacao, REML, genetic gains, SNP, breeding values, heritability

## Abstract

Breeding programs of cacao (*Theobroma cacao* L.) trees share the many challenges of breeding long-living perennial crops, and genetic progress is further constrained by both the limited understanding of the inheritance of complex traits and the prevalence of technical issues, such as mislabeled individuals (off-types). To better understand the genetic architecture of cacao, in this study, 13 years of phenotypic data collected from four progeny trials in Bahia, Brazil were analyzed jointly in a multisite analysis. Three separate analyses (multisite, single site with and without off-types) were performed to estimate genetic parameters from statistical models fitted on nine important agronomic traits (yield, seed index, pod index, % healthy pods, % pods infected with witches broom, % of pods other loss, vegetative brooms, diameter, and tree height). Genetic parameters were estimated along with variance components and heritabilities from the multisite analysis, and a trial was fingerprinted with low-density SNP markers to determine the impact of off-types on estimations. Heritabilities ranged from 0.37 to 0.64 for yield and its components and from 0.03 to 0.16 for disease resistance traits. A weighted index was used to make selections for clonal evaluation, and breeding values estimated for the parental selection and estimation of genetic gain. The impact of off-types to breeding progress in cacao was assessed for the first time. Even when present at <5% of the total population, off-types altered selections by 48%, and impacted heritability estimations for all nine of the traits analyzed, including a 41% difference in estimated heritability for yield. These results show that in a mixed model analysis, even a low level of pedigree error can significantly alter estimations of genetic parameters and selections in a breeding program.

## Introduction

*Theobroma cacao* L. is a small understory tree native to the Amazon estuary (Motamayor et al., 2008). *T. cacao* has been of cultural and economic importance for more than 2,000 years as a source of both cocoa and cocoa butter. The estimated global production of cacao was ~4 million tons in 2011–2012, with an estimated market value of between 8 and 10 billion USD (CacaoNet, 2012). Demand has increased steadily at a rate of ~3% per year, while supply has been characterized by wide fluctuations (CacaoNet, 2012). Today, 90% of the world's cacao is produced on farms smaller than 5 ha. Seventy-five percent of global production comes from West African countries, where farmers achieve a yield of 300 kg/ha on average (Lim and Pang, 1990; CacaoNet, 2012). The estimated yield potential based on the cultivation of improved clonal material in intensified farming systems has been estimated to be 2.7 t/ha, and has been demonstrated in high-density production environments in Southeast Asia and Latin America (Lim and Pang, 1990; Schnell et al., 2005; Laliberté and End, 2015). On average, an estimated 20% of worldwide production of cacao are lost annually to five major diseases: black pod (*Phytophthora* spp.), witches' broom (*Moniliopthora perniciosa*), cacao swollen shoot virus, and frosty pod/moniliasis (*Moniliophthora roreri*) (Bowers et al., 2001). Hence, the use and dissemination of improved genetic material in the form of high-yielding clones with resistance to a range of pests and diseases constitute the primary objective for future cacao breeding efforts to continue meeting demand, and buffering fluctuations in supply (Phillips et al., 2009).

Despite its global economic importance and long history of use and variety selection, established modern breeding programs for *T. cacao* have existed for less than a century and have advanced only a few generations. Most of the germplasm currently used in breeding programs worldwide has origins in material obtained during the collection expeditions of the 1930s and 1940s from seedling populations as well as clonal material. These were obtained from collections in the centers of diversity in Ecuador and Peru by F.J. Pound seeking sources of natural resistance to witches' broom (*Moniliophthora perniciosa*) (Lockwood and End, 1992; Bekele and Bekele, 1996). The earliest systematic efforts in recurrent selection aimed at a diverse base population began in Trinidad in the 1930s (Dias, 2001); these efforts involved only four parental clones (IMC 67, SCA 6, P 18, and ICS 1), and selections were based on fruit index and resistance to Ceratocystis wilt and witches' broom (Gonsalves, un published). From this rather narrow base population a series of improved genotypes were generated. In addition, strong performance of selections from other diverse crosses confirmed heterosis in cacao and established the practice of heterotic hybrids (Dias, 2001).

The primary breeding objectives for cacao have remained largely unchanged in the past 80 years for breeding programs of the crop and include the improvement of both yield, resistance/tolerance to pests and diseases, and the improvement of bean quality. In general, the two strategies utilized in cacao breeding include: (1) individual tree selection through vegetative propagation and (2) production of sexual families by open and controlled pollination (Soria, 1975). While broad diversity and the existence of heterotic groups have led to the widespread practice of hybrid seedling production, recurrent selection schemes in cacao are increasingly implemented to exploit additive and non-additive genetic variance and maximize genetic gains in the selection of high performing clones. Despite these clear objectives and consistent targets for improvement, progress made by breeding programs in cacao have fallen short of expected gains.

Particularities of cacao propagation, management and physiology give rise to unique challenges for breeding. These include, among others, the presence of genetic self-incompatibility in many populations, temporal variability in the genetic control of diverse traits and their phenotypic expression, diverse modes of vegetative and sexual reproduction, and a lack of consistent standards and methods in evaluating resistance (Dias, 2001). Furthermore, due to the labor and land-intensive requirements for long-term field evaluations, the sexual incompatibility of certain crosses, and the differential germination and survival rates of progeny, phenotypic data in cacao are often of an unbalanced nature and frequently include mislabeled individuals and measurement errors. The design and establishment of effective and successful breeding programs require detailed understanding of the genetic architecture of traits of interest, including heritability, dominance, genetic correlations, and the effects of environment, which are critical for evaluating the potential to achieve future genetic gains. The heritability of yield components and other key traits for cacao improvement are, in general, poorly understood, with most existing studies concerned with the selection and production of commercial hybrids and not the improvement of genotypes through additive genetic gain (Dias, 2001).

Linear mixed model (LMM) methodology, using restricted estimation maximum likelihood (REML) allows for reliable prediction of additive and genotypic effects (e.g., breeding values or BLUPs) despite the often unbalanced nature of phenotypic data (Resende and Dias, 2000; Dias and Resende, 2001). Best linear unbiased prediction (BLUP) values are well-suited for cacao breeding because of their maximization of selection accuracy, minimization of prediction error, and unbiased nature (Dias, 2001). LMM produces an estimation of the variance components associated with the random effects of the model, while incorporating the genetic relationship amongst all individuals into the model fit based upon a pedigree by using the numerator relationship matrix. Hence, it makes use of all available information to best estimate genetic values. Advantages of REML estimations are hindered when there are errors in the pedigree, reducing the accuracy of genetic parameter estimations, and translating into lower genetic gains (Munoz et al., 2014). The issue of pedigree errors is prevalent in other breeding programs for tree and fruit crops, as well as animals, and has been estimated to be 10%, on average (Banos et al., 2001; Visscher et al., 2002; Doerksen and Herbinger, 2010; Lacombe et al., 2013; Munoz et al., 2014). In cacao, there are opportunities to introduce pedigree errors at almost every level of the breeding program, resulting in mislabeled accessions, or “off-types.” These include the acquisition of material in the field, pollen contamination or accidental selfing, labeling mistakes, nursery mix-ups, and rootstock escape (Turnbull et al., 2004). In cacao collections, concerted efforts to identify germplasm through SNP and SSR fingerprinting and compare against referenced standards have confirmed high levels of off-types across different collections, relative to rates recorded in other species. In hybrid trials, such rates have been reported as 5.9–8.6% (Dadzie et al., 2013), 11.8% (Cervantes-Martinez et al., 2006), 30% (Schnell et al., 2005), and 54.5% (Padi et al., 2015). In seed gardens, the level of off-types amongst parents has been reported at 0–100% within a plot (Padi et al., 2015) and 35% (Olasupo et al., 2017). In clonal collections, reported rates include 6.9% Romero Navarro et al., 2017), 15–44% (Sounigo et al., 2001; Motilal and Butler, 2003), 20–100% (Padi et al., 2015), 46.4% (Aikpokpodion et al., 2009), 57.4, and 78% (Olasupo et al., 2017). The distribution and use of these individuals in breeding programs alter the expected genetic gains resulting from bi-clonal crosses, and will affect all subsequent generations when mislabeled germplasm is used in recurrent selection schemes (Adomako, 2006; Dadzie et al., 2013). In hybrid seed gardens of West Africa, the frequent use of off-type parents could be a major contributing factor to failures in meeting predicted productivity (Cervantes-Martinez et al., 2006; Padi et al., 2015). Molecular fingerprinting techniques are useful tools for the correction of labeling errors in seed gardens and germplasm plots (Livingstone et al., 2012; Takrama et al., 2014; Olasupo et al., 2017). Although it has been recommended that all parental stock be genotyped before use in breeding programs, the actual magnitude of impact that off-types have on breeding progress in cacao has never been assessed (Takrama et al., 2005; Padi et al., 2015).

In this study, we performed a multisite analysis combining data from four breeding trials situated at the Almirante and Cinco Porcos farms of the Mars Center for Cacao Science (MCCS) in Barro Preto in the state of Bahia, Brazil (14°42′45 N, 39°22′13 E). We estimated genetic parameters such as heritability, dominance ratio, genetic correlation, and genetic gain for the different traits and created for each genotype a ranked index according to weighted breeding values. The identities of all trees from a single trial (PT08) were validated with a 96 SNP off-typing marker set to determine the presence of mislabeled accessions, and genetic parameters were calculated both with and without off-types included. Hence, the two main objectives of these analyses were: (1) to increase our understanding of the genetic architecture of different traits through a multi-site analysis, and (2) to understand the impact of off-types on the estimation of genetic parameters, genetic gains and selections within a breeding program.

## Materials and methods

### Genetic material and experimental design

Genetic material from four progeny trials (PT07, PT02, PT03, and PT08) was used for this study. The trials were planted between 2000 and 2009 and evaluated from 2003 to 2015 (Table 1). The combined dataset includes 6,528 progeny from 145 bi-parental crosses made from 50 parental genotypes (Table S1). The objectives of these progeny trials were to select new parents for improved yield and sources of resistance to witches' broom, the dominant disease pressure in the region, and select trees for clonal evaluation. Although different crosses were made in the different trials, there was genetic connectivity across sites through the pedigree, with common parents, grandparents, and families shared between sites (Table S1). The parents used in trials PT07, PT02, and PT03 included international clones and local material from the germplasm collection. The trial PT08, from which our selections were made, tested seven selections from trials PT07 and PT02 as parents as well as diverse wild accessions from the germplasm collection selected for superior yield and disease resistance. Pedigree information was available for all individuals in the trials and included 6,592 individuals: 6,528 offspring and 64 parents, grandparents, and great-grandparents. The parents of all individuals in the progeny trials, and grandparents when available, are listed in the pedigree file (Table S1).

**Table 1:** Summary of details of progeny trials considered in the multisite analyses.

**Trial**	**PT07**	**PT02**	**PT03**	**PT08**
Planting date	05/2000	04/2001	04/2004	12/2009
Measurement years	2003–2009	2003–2007	2008–2015	2011–2015
Crosses	17	12	51	69
Parents	18	11	13	23
Progeny	714	322	1,946	3,546
Reps	4	2	4	3
Trees/ Plot	20	10	10	18
Area (ha)	1.73	0.46	1.75	2.1
Spacing (m × m)	3 × 3	3 × 3	3 × 2.5	2.5 × 2.5

### Phenotypic information

Phenotypic data were collected on yield components and resistance to pests and diseases. Yield (Yd, kg/ha/yr) was defined as total dry bean weight, obtained from monthly wet bean weight measurements converted to dry bean weight, by multiplying with the coefficient of 0.4 (Are and Atanda, 1972). This measurement of dry bean weight per tree was translated into an estimated yield/ha by multiplying the mean yield per plant by 1,111 (equivalent to a spacing of 3 × 3 m) to allow for a comparison of yield across trials. The seed index (SI, gr) was calculated as the dry weight of 100 seeds, recorded monthly. The pod index (PI, pods/kg) was calculated monthly from the number of healthy pods required to obtain 1 kg of dry bean weight. Percent healthy pods (%HP, %) was a measure of the percentage of healthy pods discounting pods with disease as well as pest damage, calculated monthly. The percentage of *Phytophthora*-infected pods (%PP) was a cumulative measure of pod loss due to black pod rot (*Phytophthora* spp.), calculated monthly. The percentage of witches' broom-infected pods (%WBP), a measure of pod loss due to witches' broom (*M. perniciosa*), was calculated monthly. The percentage of pods due to other losses (%OL) is a ratio of the pods lost to other stresses besides *Phytophthora* spp. and witches' broom, including pest damage from rats and birds, and was also measured once per month. The vegetative brooms (VB) parameter included a count of vegetative brooms per tree caused by witches' broom; these were counted and removed on an annual basis. All of the above traits were measured in all trials, except vegetative brooms that was not obtained for trial PT03, and seed index, which was not obtained for trial PT07. For trial PT08 only, measures of vigor were included when the trees reached 6 years of age. This consisted of stem diameter (D, cm) measured for each tree at a height of 30 cm aboveground, and height (Ht, m) measured as the distance from the base of the tree to the first jorquette. Note that cacao plants follow a dimorphic growth form, exhibiting orthotropic development until a certain developmental stage when they produce a jorquette and develop plagiotropic fan shoots.

### Multisite analysis

Multisite analyses incorporate information from several sites to obtain overall breeding values and reduce bias in estimates of heritability by accounting for genotype by environment (G × E) interactions on different traits. The four trials included in the multisite analysis are located within the two farms of MCCS, and were evaluated during different time intervals between 2003 and 2015 (Table 1). Residuals for all response variables were checked for normality and heterogeneity of variances and no important departures were noted. The only trait that required transformation was yield, which was implemented as log(yield/100 + 1) ^*^ 10. All the data were analyzed jointly in the multisite analysis fitted using the following LMM:

$$\begin{array}{l} y\;=\;\mu 1+X_{\text{1}}s+X_{\text{2}}bs+Z_{\text{1}}a+Z_{\text{2}}as+W_{\text{1}}f+W_{\text{2}}fs+W_{\text{3}}ps+e \end{array}$$

where ***y*** is the vector of the response variable; μ is the overall mean effect; ***s*** is the fixed vector of site effects; ***bs*** is the fixed effects vector of block-within site; ***a*** is the random vector of individual effects representing specific combining ability, with ***a*** ~ MVN(**0**, $\sigma_{\text{a}}^{2}$***A***); ***as*** is the random effects vector of interaction of individuals with site, with ***as*** ~ MVN(**0**, $\sigma_{\text{as}}^{2}$***I***_*as*_); ***f*** is the random effects vector of family, with ***f*** ~ MVN(**0**, $\sigma_{\text{f}}^{2}$***I***_*f*_); ***fs*** is the random effect vector of interaction of family with site, with ***fs*** ~ MVN(**0**, $\sigma_{\text{fs}}^{2}$***I***_*fs*_); ***ps*** is the random effects vector of plot within-site, with ***ps*** ~ MVN(***0***, ***D***_*ps*_); and ***e*** is the random vector of errors, with ***e*** ~ MVN(**0**, ***D***_*e*_). ***X***_*1*_, ***X***_*2*_, ***Z***_***1***_, ***Z***_***2***_, ***W***_***1***_, ***W***_**2**_, and ***W***_***3***_ are all incidence matrices, ***1*** is a vector of ones and ***I*** are identity matrices of their corresponding size. The matrix ***A*** is the numerator relationship matrix for individuals calculated using the corrected pedigree (described under Single Site Analysis). ***D***_*ps*_ and ***D***_*e*_ are block diagonal matrices where the different sites have their own independent *ith* plot and error term component, $\sigma_{\text{psi}}^{2}$ and $\sigma_{\text{ei}}^{2}$, respectively.

Narrow-sense heritability was calculated as $h^{2}={\sigma_{a}}^{2}/\left({\sigma_{a}}^{2}+{\sigma_{as}}^{2}+{\sigma_{f}}^{2}+{\sigma_{fs}}^{2}+{\bar{\sigma_{p}}}^{2}+{\bar{\sigma_{e}}}^{2}\right)$ and the dominance ratio was calculated as $\;d^{2}\;=4{\sigma_{f}}^{2}/\left({\sigma_{a}}^{2}+{\sigma_{as}}^{2}+{\sigma_{f}}^{2}+{\sigma_{fs}}^{2}+{\bar{\sigma_{p}}}^{2}+{\bar{\sigma_{e}}}^{2}\right)$, with σ_*a*_^2^ being the additive variance, σ_*as*_^2^ the genotype within site variance, σ_*f*_^2^ the family variance, σ_*fs*_^2^ the family within site variance, ${\bar{\sigma_{p}}}^{2}$ the mean of plot variance across the four sites, and ${\bar{\sigma_{e}}}^{2}$ the mean error variance across the four sites. Also, a Type-B (r_B_) additive genetic correlation across the four sites was calculated as $r_{B}={\sigma_{a}}^{2}/{\left({\sigma_{a}}^{2}+{\sigma_{as}}^{2}\right)}_{.}$ All variance components and genetic parameters were estimated by fitting the previously described LMM using the library ASReml-R 3.0 (Gilmour et al., 2009) for the R statistical package (R Core Team, 2013).

### Selection of genotypes

The estimated breeding values (BV), which represent deviations from the overall mean phenotypic values, were obtained from the multisite analysis described above. These were later converted into predicted values by adding to each BV an overall phenotypic mean. In the case of yield, a back-transformation was implemented over these predicted values to express it in its original units. Finally, for each of traits predicted values were standardized (i.e., Z-scores) for the construction of an index for clonal selection. Here, economic weights were assigned to each trait with the primary objective of advancing yield and a second objective of increasing resistance to witches' broom. This index was constructed using the standardized values of the traits Yd, PI, and VB with weights equal to 0.7, −0.15, and −0.15, respectively. The negative values are required to decrease the response of PI and VB. The genetic gains from selections were calculated as the ratio of the mean phenotype plus the breeding value for the top 1 and 5% of individuals for each trait divided by the phenotypic mean plus the overall average breeding value.

### Evaluation of off-types

Leaf punch samples were collected from all trees in the field and outsourced to LGC Genomics (Middlesex, UK) for fingerprinting by KASP™ genotyping. A 96 SNP offtyping marker set was used to fingerprint all progeny and parents used in PT08. This marker set can reliably distinguish between closely related individuals, and has been used widely for offtyping in cacao breeding programs (Allegre et al., 2012; Padi et al., 2015; Table S2). A genotypic analysis was run using PLINK (Purcell et al., 2007) to scan the dataset for errors (using the –mendel command) and identify progeny that did not follow the expected segregation for their given family, while parents were compared against each other clonal replicates in the germplasm collection and also against international reference standards (Dapeng Zhang, personal communication).

A single-site analysis of PT08 was fitted to determine the effect of off-types on estimation of genetic parameters. The single-site analysis used a similar structure as the multisite analysis with the following LMM:

$$\begin{array}{l} y=\mu 1+Xb+Za+W_{\text{1}}f+W_{\text{2}}p+e \end{array}$$

where ***y*** is the vector of the response variable; μ is the overall mean effect; ***b*** is the fixed effects vector of block; ***a*** is the random effects vector of individual effects representing specific combining ability, with ***a*** ~ MVN(**0**, $\sigma_{a}^{2}$***A***); ***f*** is the random effects vector of family, with ***f*** ~ MVN(**0**, $\sigma_{\text{f}}^{2}$***I***_*f*_); ***p*** is the random effects vector of plot, with ***p*** ~ MVN(**0**, σ_*p*_^2^***I***_*p*_); and ***e*** is the random vector of errors, with ***e*** ~ MVN(**0**, $\sigma_{\text{e}}^{2}$***I***_*e*_). ***X***, ***Z***, ***W***_*1*_ and ***W***_*2*_ are incidence matrices. All other terms were previously defined.

The single-site models from PT08 were fit first with all individuals, and then with off-types removed through the pedigree to determine the impact of these individuals on the estimation of genetic parameters. Later, genetic gains were obtained by dividing the predicted values for each genotype by the trial average, and were calculated using a 1% selection intensity according to the weighted index described earlier. Genetic gains were calculated first from the complete dataset of all individuals and then the dataset with off-types excluded.

In addition, for the PT08 data, trait-to-trait, or type-A (r_A_) additive genetic correlations were estimated by running a pairwise bivariate analysis model across the different pairs of traits. This was based on a model that considered the same terms as above but, in addition, a heterogeneous correlation structure was considered for the all random effects, including the error term. Standard errors of heritability, dominance ratio and genetic correlations were approximated using the delta method.

The proportion of membership to each of the 10 cacao genetic groups was estimated using Admixture software (Alexander et al., 2009). A supervised admixture analysis was performed using individuals with over 85 proportion ancestry from the 10 cacao ancestral groups (Motamayor et al., 2008). Proportion of ancestry was analyzed for the individuals with the top 1% BLUP breeding values for each trait and compared against the population mean to determine which genetic backgrounds were over represented for some traits.

## Results

### Genetic architecture

A summary of phenotypic traits for each trial is provided in Table 2. In relation to all yield components, mean yield (Yd) showed the largest variation between trials, ranging from 93.7 kg/ha/yr (PT07) to 370.6 kg/ha/yr (PT02), while seed index (SI) ranged from 133 g (PT02) to 142.3 g (PT03) in the trials for which it was measured. Pod index (PI) ranged from 24.3 pods (PT03) to 29.7 pods (PT07). *Phytopthora-*infected pods (%PP) was low across all trials, ranging from 3.1 to 7.7%, while witches' broom pods (%WBP) ranged from 5.7% in PT02 to over 20% in PT03 and PT08. Pods lost from other pests and diseases (%OL) was also higher in the two most recent trials (PT03 and PT08). The higher pest and disease incidence is reflected in the percentage of healthy pods (%HP), which was more than 80% for PT07 and PT02 but the percentage decreased to 53.6 and 56.8% for PT03 and PT08, respectively. The trial average of vegetative brooms (VB) removed annually ranged from 1.3 (PT07) to 5.9 (PT02). Differences in phenotypic values between the different trials reflect differences in the families tested, trial size, and duration as well as site-specific effects such as differences in disease pressures, and helped to provide measures of G × E on the traits evaluated (Table 2).

**Table 2:** Phenotypic means and standard deviations for evaluated traits for all trials and in each trial.

**Trait**	**All**	**PT07**	**PT02**	**PT03**	**PT08**
Yd	183.9 (209.6)	93.7 (128.9)	370.63 (329.8)	128.17 (144.1)	215.72 (220.8)
SI	137.3 (30.7)	–	133.0 (35.1)	142.3 (32.2)	135.5 (29.2)
PI	26.8 (10.1)	29.7 (13.4)	24.7 (7.7)	24.3 (9.0)	27.8 (9.9)
%HP	59.6 (23.4)	81.1 (20.4)	83.8 (15.9)	53.6 (21.2)	56.8 (22.0)
%PP	4.5 (9.1)	7.7 (13.7)	6.4 (10.3)	5.8 (9.0)	3.1 (7.6)
%WBP	20.0 (17.5)	9.2 (14.5)	5.7 (8.7)	22.4 (16.7)	21.9 (17.7)
%OL	15.6 (16.0)	1.9 (8.0)	4.1 (8.8)	17.4 (15.2)	18.1 (16.2)
VB	3.2 (4.6)	1.3 (4.3)	5.9 (8.1)	–	3.3 (4.0)
D	–	–	–	–	79.1 (17.7)
Ht	–	–	–	–	1.2 (3.6)

*Yd is yield, measured as kg/ha/yr; SI is seed index, the dry weight of 100 beans; PI is pod index, the number of pods to reach 1 kg; %HP is percent healthy pods; %PP is percent of pods infected by phytophthora; %WBP is percent of pods infected with witches' broom; %OL is percent of pods lost to other pressures such as pests; VB is the count of vegetative brooms removed each year; D is diameter (cm) at 30 cm; and Ht is height of first jorquette (m)*.

Narrow-sense heritability estimates differ greatly between sites and traits (Table 3). The multisite analysis resulted in an estimated narrow-sense heritability that ranged from 0.05 for %OL to 0.64 for PI (Table 3). PT08, the largest trial with the most parents and crosses, represented 56% of the total progeny evaluated and consistently had a higher heritability for most traits with the exception of %HP and %WBP. The highest heritability was found for the trait PI across all sites, followed by Yd. The prevalence of *Phytopthora* was determined to be too low across sites to provide accurate measures of heritability and correlations, so the trait %PP was excluded from the analyses of genetic architecture.

**Table 3:** Narrow-sense heritability and dominance ratio estimates (with standard errors) for multiple site (MET) and single site analyses and estimated genetic gains on PT08.

**Trait**	**MET**		**PT07**		**PT02**		**PT03**		**PT08 (with off-types)**		**PT08 (without off-types)**		**PT08 genetic gains**	
	***h^2^***	***d^2^***	***h^2^***	***d^2^***	***h^2^***	***d^2^***	***h^2^***	***d^2^***	***h^2^***	***d^2^***	***h^2^***	***d^2^***	***Top 1%***	***Top 5%***
Yd	0.37 (0.01)	0.00 (0.00)	0.44 (0.17)	0.00 (0.00)	0.21 (0.28)	0.23 (0.40)	0.15 (0.09)	0.30 (0.09)	0.35 (0.12)	0.80 (0.17)	0.59 (0.11)	0.39 (0.11)	0.93	0.76
SI	0.63 (0.01)	0.00 (0.00)	–	–	0.23 (0.40)	0.01 (0.83)	0.30 (0.09)	0.55 (0.18)	0.68 (0.14)	0.04 (0.03)	0.61 (0.1)	0.04 (0.03)	0.41	0.31
PI	0.64 (0.01)	0.06 (0.02)	0.44 (0.17)	0.00 (0.00)	0.82 (0.29)	0.09 (0.15)	0.98 (0.20)	0.09 (0.03)	0.42 (0.11)	0.07 (0.03)	0.71 (0.11)	0.03 (0.03)	−0.54	−0.45
%HP	0.16 (0.00)	0.00 (0.00)	0.00 (0.00)	0.00 (0.00)	0.09 (0.1)	0.00 (0.00)	0.28 (0.11)	0.07 (0.04)	0.16 (0.06)	0.04 (0.04)	0.17 (0.06)	0.04 (0.04)	0.22	0.19
%WBP	0.15 (0.00)	0.00 (0.00)	0.01 (0.05)	0.02 (0.08)	0.11 (0.10)	0.00 (0.00)	0.26 (0.10)	0.02 (0.03)	0.14 (0.05)	0.08 (0.04)	0.15 (0.05)	0.07 (0.04)	−0.49	−0.39
%OL	0.05 (0.00)	0.07 (0.03)	0.01 (0.03)	0.00 (0.00)	0.00 (0.00)	0.00 (0.00)	0.08 (0.05)	0.11 (0.06)	0.10 (0.04)	0.05 (0.03)	0.13 (0.05)	0.05 (0.04)	−0.36	−0.30
VB	0.03 (0.00)	0.00 (0.00)	0.08 (0.14)	0.22 (0.23)	0.10 (0.23)	0.24 (0.33)	0.08 (0.00)	0.30 (0.00)	0.22 (0.07)	0.02 (0.03)	0.16 (0.05)	0.02 (0.04)	−0.62	−0.57
D	–	–	–	–	–	–	–	–	0.11 (0.05)	0.08 (0.06)	0.12 (0.05)	0.04 (0.05)	0.10	0.08
Ht	–	–	–	–	–	–	–	–	0.34 (0.09)	0.01 (0.03)	0.27 (0.07)	0.00 (0.00)	0.29	0.23

*For PT08 heritabilities and dominance ratio estimates are presented with and without off-types*.

Understanding genetic correlations between sites (r_B_; type-B correlations) provides a measure of G × E interaction on the trait. Type-B correlation values range from −1 to 1, with scores close to ±1 indicating high stability across environments. Type-B additive correlations were high across all traits, and yield-related traits in particular were extremely stable, ranging from 0.81 (%HP) to 1 (SI). This corresponds to the high heritabilities of the different yield components. GxE was highest for witches' broom-related traits, which had lower stability across sites. Type-B correlations ranging from 0.37 for VB to 0.76 for %WBP.

An understanding of genetic correlations between traits (r_A_; type-A correlations) provides a measure of pleiotropy and can be useful for indirect selection of correlated traits. There existed a strong genetic correlation between SI and PI (−0.81), but much lower correlations between both of these traits and Yd (−0.25 ± 0.17 and −0.12 ± 0.18). The weighted index used in our selections included PI and Yd as the yield components. This would allow for indirect selection of SI based on the strong correlation with PI. The low correlations between yield components and total yield support the inclusion of overall yield and an index (pod or seed index) within a breeding program. The strong correlation (−0.72) between %HP and %WBP suggests that witches' broom is driving most of the disease related losses in the trial, relative to %OL (−0.64). The correlation between %WBP and VB, which represent different stages of the same infection was only 0.27, with a large standard error. Of the two measures, %WBP was more informative of the impact to productivity, with a correlation of −0.61 with Yd and −0.72 with %HP. Of the vigor measures, D had low correlations with all other measures, including Ht. The trait Ht had strong correlations with %WBP (−0.5), Yd (0.39), and PI (0.37). This could correspond to more productive surface area along the main trunk for pod growth in cacao, a cauliflorous crop. The trait %HP showed strong genetic correlations with both yield components and disease related traits, despite having a low heritability itself.

### Selection of genotypes and genetic gains

Weights and standardized BVs from the multisite analyses were used to construct a weighted index by which to select the top 1% of individuals from PT08 for clonal evaluation. Of the 69 crosses and 3,458 individuals, a small number families were over-represented in the ranked selections. Of the top 100 individuals, 46 came from only four families, with 20 from the cross GU-144C × MC-08017, 10 from the cross GU-144C × MC-08091, nine from the cross MC-08005 × MC-08060, and seven from the cross CCN-51 × MC-08017. Notably, CCN-51 is a parent of each of the MC selections from previous trials represented here. Genetic gains in PT08, based on the BLUP values of the top 1% individuals for each trait, included a 93% increase for yield, 62% reduction in vegetative brooms, a 54% reduction in pod index, and a 40% increase in seed index (Table 3). The population structure analysis showed a large degree of admixture from the different genetic groups, with Amelonado, Criollo, and Iquitos present in the highest proportion (Figures 3A,B). The large proportion of Amelonado and Criollo ancestry is attributable to the high prevalence of CCN-51 in the pedigree.

### Evaluation of off-types

**Figure 1:**
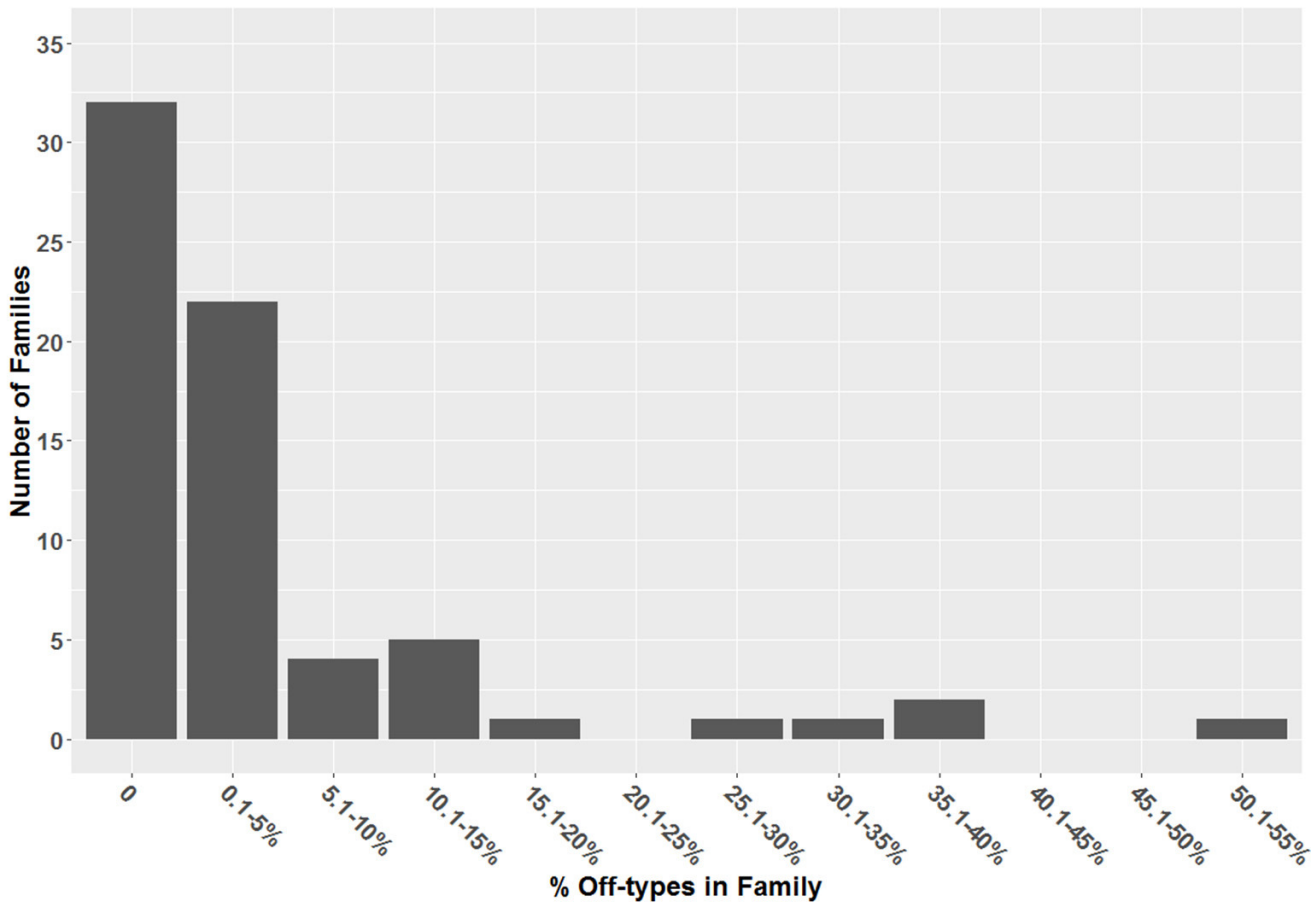
The number of families corresponding to different proportions of off-types in PT08.

**Figure 2:**
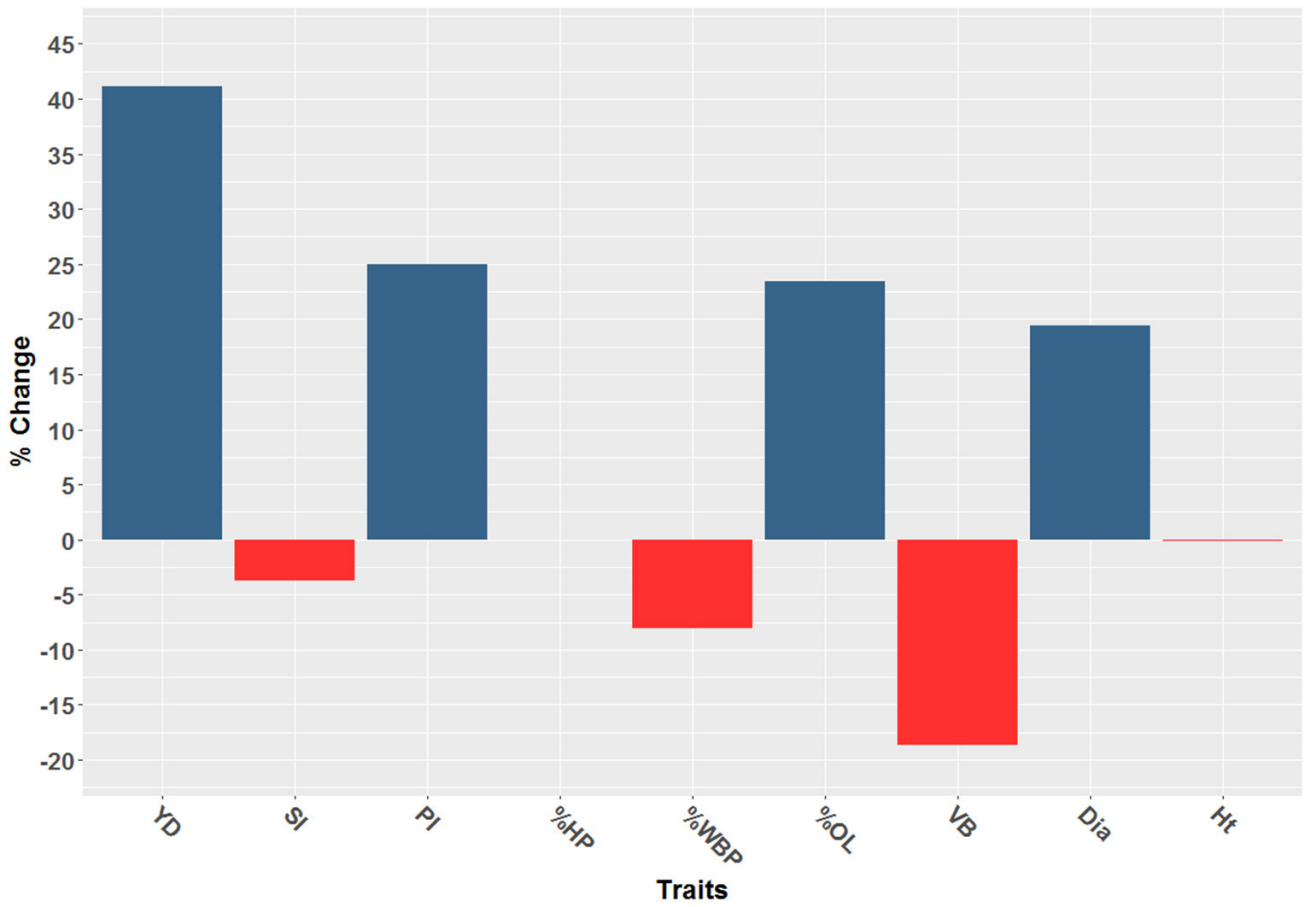
The percent change in genetic gains by trait resulting from the exclusion of off-types in the single site analysis. For blue traits, the estimated gains increase once off-types are removed but for red traits, the estimated gains decrease with the removal of off-types.

A total of 166 individuals (4.7%) out of the 3,516 individuals of PT08 were classified as off-types (Figure 1). The inclusion of off-types in the analysis resulted in changes to the estimations of heritability for all nine of the traits, and these impacts varied considerably by trait (Figure 2). The inclusion of off-types in the pedigree deflated the heritability estimation for Yd by 41%, and increased the estimation of dominance by 134%. The off-type data set presented 33% lower heritability for PI compared with the data set in which they were excluded, while *d*^2^ was relatively unaffected. The narrow-sense heritability for VB was inflated by 25% in the dataset that included off-types (Table 3). One important effect of the detection of off-types is that, while constituting just 4.7% of the population of individuals, there was a 48.6% change in top 1% selections, translating into major changes in current and future genetic gains. Removal of off-types increased estimated genetic gains on Yd by 34%, and PI by 23%. Estimated genetic gains for VB and %WBP traits actually decreased with the removal of the off-types, with a reduction of 15% for VB, for example (Figure 2). The differences in magnitude and direction of the genetic gains estimations demonstrate the high levels of error introduced into BLUP based selection models as a result of pedigree errors. Notably, when the parents of the trial were fingerprinted for the off-type analysis, it was observed that none of the NA-33 clonal accessions matched the international reference genotype provided by the ICGD, and thus the tree represented a monotypic off-type in the collection. Results of a genetic population structure analysis demonstrate that the tree NA-33 in the MCCS germplasm collection is not a pure Nanay, but represented population admixture between Nanay (61.5%), Iquitos (22.6%), and <10% contribution from Contamana, Purus, Maranon, and Criollo (Table S2).

## Discussion

In this study, four trials were analyzed in a multisite analysis to evaluate the genetic architecture of 10 traits, and make selections from the trial PT08 using a weighted index. Also, a single-site analysis was performed for trial PT08 after using molecular markers to identify and remove off-types to determine the magnitude of impact that pedigree errors have on the estimation of genetic architecture. Our findings build upon previous studies in cacao and other crops and will be relevant for informing future breeding strategies for cacao. In addition, we identify both promising selections for clonal testing, and promising potential sources of resistance to witches' broom.

Few prior studies have established narrow-sense heritability estimations for cacao. In our multisite analysis, pod index (PI) exhibited the highest heritability of all traits evaluated (0.64 ± 0.1), and showed a high stability across sites as well (0.96 ± 0.05). It had a high genetic correlation with seed index (−0.81 ± 0.07), and only a moderate correlation with yield (−0.25), making both traits good targets for selection. The heritability obtained for PI falls between previous reported heritabilities of 0.14 (Ofori et al., 2016) and 0.88 (Opoku et al., 2010); also, the influence of dominance was very low (0.03–0.09) on this trait. Individuals with the best BV estimations for this trait came predominantly from crosses with CCN-51 in the genetic background. The progeny with the highest BVs for PI came from crosses that included CCN-51, MC-08024, a selection from the cross CCN-51xTSH-565 present in PT02 (S1), and the parent Ipiranga-1.

**Figure 3:**
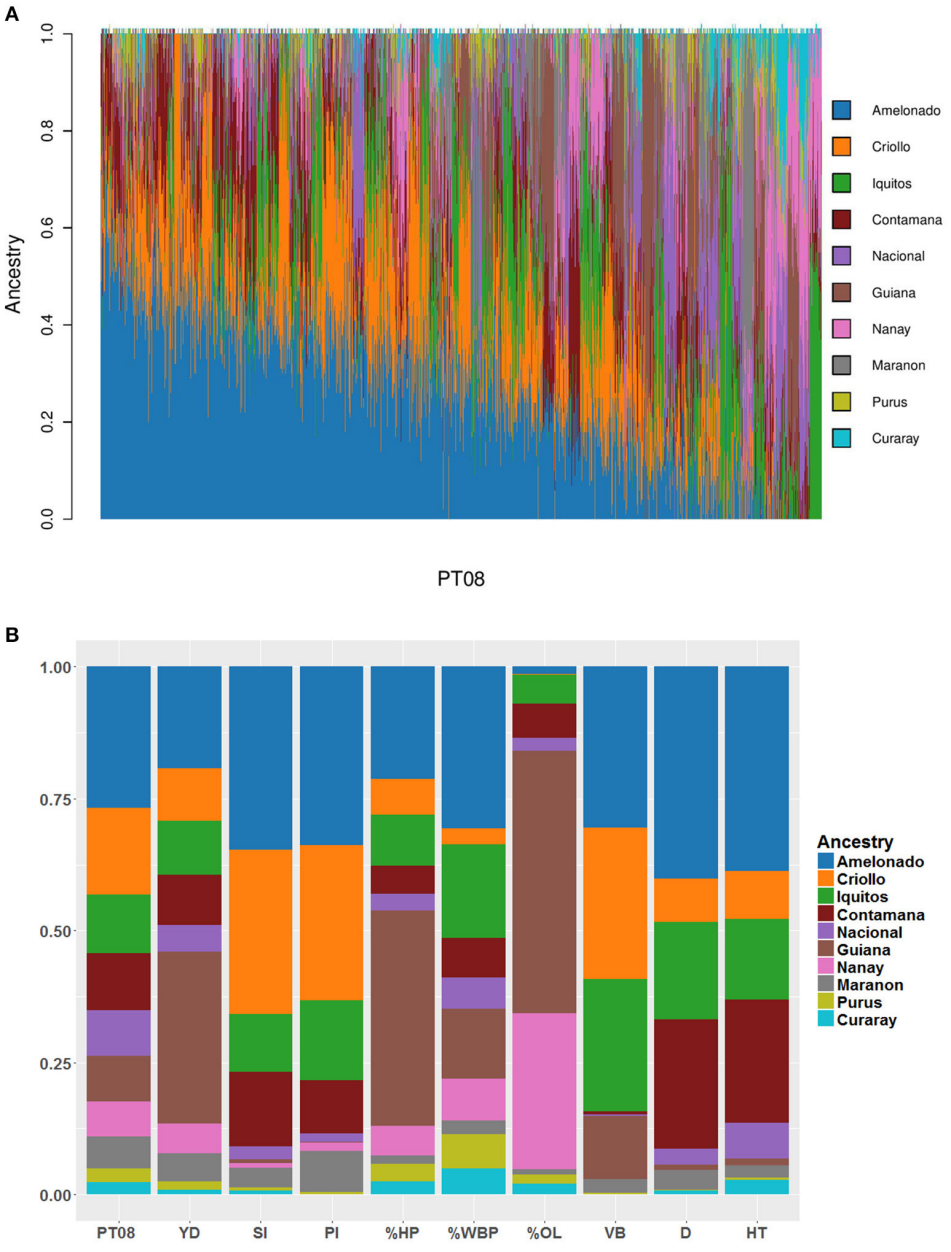
**(A)** Population structure of 3,358 progeny from the trial PT08. **(B)** The proportions of ancestry in the genetic background of individuals with the top 1% breeding values per trait.

For yield the multisite analysis estimated an heritability of 0.37 ± 0.01, which is higher than the range reported in previous studies, including 0.11 ± 0.1 (Padi et al., 2015), and 0.17 reported for fresh bean weight over 3 years (Soria, 1974), and falls within the broad-sense heritability values of 0.15 (Lockwood et al., 2007), 0.18 ± 0.4 (Ofori et al., 2016), and 0.42 (Opoku et al., 2010) (Table 3). In our study, the influence of dominance variance on yield was particularly strong in all trials, supporting previous reports of heterosis for the crop (Atanda, 1972; Lachenaud et al., 2007). Progeny with the highest breeding values for yield corresponded with a higher contribution of Guiana ancestry in their genetic background (Figure 3B). This group represents a genetically and geographically isolated wild subpopulation of cacao (Motamayor et al., 2008) and also supports the importance of exploiting heterosis in cacao breeding. Guiana ancestry was also high in progeny with the best breeding values for %HP, %WBP, and %OL, suggesting a role in disease resistance as well. Good predicted performance for the trait %OL corresponds also with a strong representation of the Nanay subpopulations (Figure 3B).

The highest heritability of the disease resistance traits in the MET was in %WBP (0.15 ± 0), and showed high stability across sites as well (0.76 ± 0.22). PT08 progeny with the top 1% BVs for this trait all came from a single cross between two PT02 selections: MC-08005, a cross between CCN-51 and GU-171c, and MC-08047, a cross between IP-128 and COCA-3370/5. On average, the genetic gain for trees from this cross showed a 47% reduction in %WBP compared to the trial mean. Vegetative brooms showed a low narrow-sense heritability in the multisite analysis and low stability, suggesting a strong effect of environment. High variability in vegetative brooms have been demonstrated in field trials, ranging from 0 to 90 brooms (Pires et al., 1999). Our results differ from the findings of Lopes et al. (2003), who reported that witches' broom resistance had a higher heritability than yield. Previous observations of segregating families led to conclusions about the oligogenic inheritance of witches' broom resistance (Soria, 1977; Royaert et al., 2016), and our findings appear to support the involvement of multiple genetic sources of resistance. A promising source of resistance from this trial came from the combination of CCN-51xNA-33 in the pedigree, with 76 of the 100 top-ranked progeny for this trait possessing this parentage either as CCN-51xNA-33 or as a backcross of a selection from PT02 (CCN-51xMC-08017) with CCN-51. The genetic gain of trees in PT08 with this combination in the pedigree showed a 12% genetic gain in the reduction of witches' broom.

Four crosses accounted for 66 of the top 100 progenies selections after the weighted index was applied; GU-144CxMC-08017, GU-144CxMC-08091, and MC-08005xMC-08060. This compares favorably with other studies, where a small number of families out-yield the average cross (Opoku et al., 2010). Stability, presented as type-B correlations, was high for yield related traits Yd, SI, PI, and %HP, while disease related traits showed greater variability across environments (Table 4). Our findings were consistent with previous work showing low GxE on yield related traits (Opoku et al., 2010). In cacao it has been documented that the highest ranking families for yield are consistently the top performing, even when tested in different years and different locations (Dias and Kageyama, 1995; Adomako and Adu-Ampomah, 2003; Opoku et al., 2010).

**Table 4:** Type-B (r_B_) additive genetic correlations between sites based on the multisite analysis, and type-A (r_A_) additive genetic correlation for single-site analysis based on the single site analysis for PT08 with offtypes excluded.

**Site correlations (r**_**B**_**)**		**Trait correlations (r**_**A**_**)**								
		**YD**	**SI**	**PI**	**%HP**	**%WBP**	**%OL**	**VB**	**D**	**Ht**
YD	0.83 (0.18)	1								
SI	1.0 (0.0)	−0.25 (0.17)	1							
PI	0.96 (0.05)	−0.12 (0.18)	−0.81 (0.07)	1						
%HP	0.81 (0.2)	0.65 (0.14)	−0.66 (0.16)	0.80 (0.12)	1					
%WBP	0.76 (0.22)	−0.61 (0.16)	0.31 (0.21)	−0.36 (0.22)	−0.72 (0.13)	1				
%OL	0.55 (0.34)	−0.32 (0.22)	0.79 (0.13)	−0.94 (0.06)	−0.64 (0.17)	−0.14 (0.28)	1			
VB	0.37 (0.57)	0.0 (0.23)	0.19 (0.21)	−0.21 (0.22)	0.01 (0.25)	0.27 (0.23)	−0.38 (0.23)	1		
D	–	−0.01 (0.25)	−0.25 (0.24)	0.06 (0.27)	0.02 (0.29)	−0.24 (0.28)	0.20 (0.28)	−0.35 (0.26)	1	
Ht	–	0.39 (0.19)	−0.12 (0.19)	0.37 (0.18)	0.35 (0.21)	−0.50 (0.19)	−0.01 (0.25)	−0.12 (0.22)	0.12 (0.25)	1

Given the cost of phenotyping large populations, the correlations found in this analysis could help reduce the traits that are collected by making use of genetic correlations for indirect selection. We found the most informative traits for indirect selection to be Yd, PI, or SI, %HP, %WBP, and Ht, and less informative phenotypic measures to be D and VB (Table 4).

Pedigree errors, mislabeling, and mistakes have been reported in breeding programs for many perennial species such as sugarcane, oil palm, and conifers among others (Adams et al., 1988; McIntyre and Jackson, 2001; Corley, 2005; Grattapaglia and Resende, 2011). When reported, effects on the estimations of genetic architecture have been relevant. In *Pinus sylvestris* L., as little as 0.5% of trees with pedigree errors significantly altered additive variance and heritability estimates; after pedigree errors reached 2%, estimates diverged beyond the expected parameter space of the genetic model (Ericsson, 1999). The impacts of mislabeled trees in breeding trials for cacao have only recently been taken into account, and in some studies poor performance and differences in girth were attributed to off-types (Ofori et al., 2012; Padi et al., 2015). Although off-types have been reported as an important issue in germplasm collections, this is the first report on the impact of off-types on key parameters for a breeding program.

In summary, we found the level of off-types in our study (4.7%) to be much lower than any of the reports in the cacao literature. Nonetheless, this still resulted in altering index selections by 48%, and altering expected genetic gains across all traits included in the analysis from 2% (%HP) to 34% (Yd). At the time of publication, records were not available on the origins of the NA-33 in the germplasm collection at MCCS; however, a literature search revealed a 99.7% genetic similarity based on RAPD markers between NA-33 held at BAL plantation in Malaysia, suggesting that the off-type may be widespread in global collections (Figueira, 1998). The discovery that a member of the pedigree of nearly all top ranking progeny for vegetative broom resistance was an off-type underscores the importance of fingerprinting parents and comparing them against reference genotypes where available.

While the applications of mixed models and BLUP estimations using REML can greatly improve estimations in cacao breeding, this study shows that uncorrected pedigree errors can alter estimated BLUPs, heritabilities, and genetic gains, for diverse traits, and impede breeding progress by altering selections. The failure of selections to meet expectations can affect farmer confidence and ultimately impede the adoption and use of molecular tools and technologies in breeding (Padi et al., 2015). Today, improvements in costs and technologies have made fingerprint verification through molecular markers possible for many breeding programs. Dense marker panels are now widely used to infer relationships in breeding programs with complex pedigrees (Powell et al., 2010; Munoz et al., 2014), but fingerprinting using small numbers of SNP reference markers (e.g., <100), can still enable the verification at a lower cost before using individuals for crosses. Genetic fingerprinting using dense marker panels for pedigree correction is not an option for most breeders. However, small panels of ~100 SNP markers can still detect deviation from the expected distributions of full-sib families and provide an indication of families that are likely to have off-types. Based on the prevalence of off-types in cacao populations and their substantial impact on breeding progress, predictive ability and selections, it is strongly advised that small off-typing marker sets available for cacao be used to validate the genetic identities at all stages of recurrent breeding programs.

## Data archiving statement

All relevant data are within the paper and supplementary files.

## Author contributions

JM, SG, and AD: designed the analysis; AD: wrote the manuscript; SR and J-PM: oversaw the collection of field data and AD, SG, DL, CS, and GM: analyzed the data. All authors offered revisions and approved the final manuscript.

### Conflict of interest statement

The authors declare that the research was conducted in the absence of any commercial or financial relationships that could be construed as a potential conflict of interest.

## Supplementary material

Table S1 Pedigree of trees including off-types in PT08.

Table S2 96 SNP off-typing markers for cacao.
